# Recent advancements in molecular photoacoustic tomography

**DOI:** 10.1088/2515-7647/adf167

**Published:** 2025-07-28

**Authors:** Eric Hall, Chengyun Tang, Lei Li

**Affiliations:** 1 Department of Electrical and Computer Engineering, Rice University, Houston, TX, United States of America; 2 Texas A&M School of Engineering Medicine, Houston, TX, United States of America; 3 Department of Bioengineering, Rice University, Houston, TX, United States of America

**Keywords:** photoacoustic tomography (PAT), photoacoustic microscopy (PAM), molecular photoacoustic tomography, molecular imaging, endogenous contrast, exogenous contrast

## Abstract

Photoacoustic tomography (PAT) is an emerging biomedical imaging technology that combines the molecular sensitivity of optical imaging with the spatial resolution of ultrasonic imaging in deep tissue. Molecular PAT, a subset of PAT, takes advantage of the specific absorption of molecules to reveal tissue structures, functions, and dynamics. Thanks to the high sensitivity to the optical absorption of molecules, PAT can selectively image those molecules by tuning the excitation wavelength to each target’s optical absorption signature. PAT has imaged various molecular targets *in vivo*, ranging from endogenous chromophores, e.g. hemoglobin, melanin, and lipids, to specialized exogenous contrasts such as organic dyes, genetically encoded proteins, and nano/microparticles. Each molecular contrast hosts inherent advantages. Endogenous contrasts allow for truly noninvasive imaging but cannot attain high specificity or sensitivity for many biological processes, whereas artificial exogenous contrasts can. Recent advances in imaging these contrast agents have shown the immense potential of photoacoustic imaging for diagnosing, monitoring, and treating medical conditions, along with studying the fundamental processes *in vivo*.

## 1 Introduction

Photoacoustic tomography (PAT), also known as optoacoustic tomography, is an emerging biomedical imaging technology that inherits the advantages of optical imaging and ultrasound (US) imaging and offers both high molecular sensitivity and high resolution in deep tissue [1, 2]. Based on the photoacoustic effect, where absorbed light is converted to sound, PAT has enjoyed rapid development in the past two decades [3].

**Figure 1:**
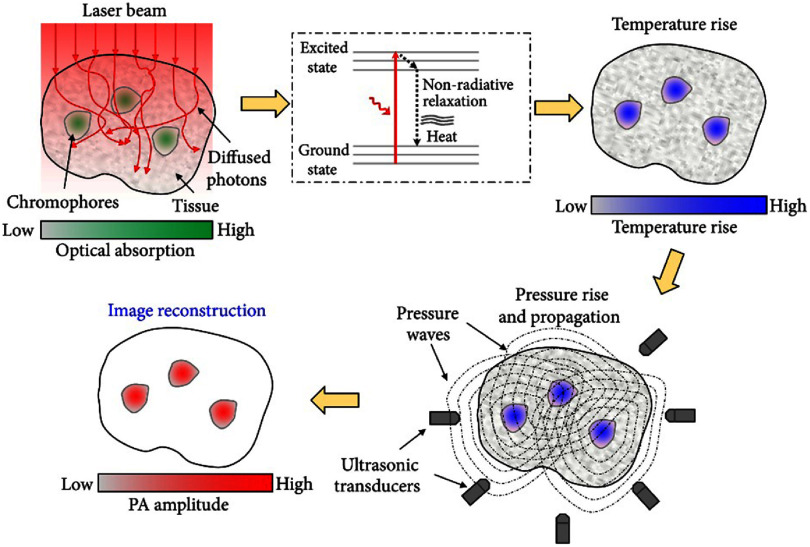
Overview of the photoacoustic effect and its use in a simplified photoacoustic tomography (PAT) imaging system. Reproduced from [2]. CC BY 4.0.

PAT typically utilizes nonionizing, pulsed laser light directed at the target to generate photoacoustic signals. Microwaves and radio frequencies can also be used for this purpose. However, this is typically called thermoacoustic tomography [4]. Incident light photons from the laser are absorbed by molecules within the target, exciting them to the excited states. When the molecules relax back to the ground states, heat is emitted after undergoing the process of nonradiative relaxation [5]. Thermoelastic expansion from the heat causes a pressure wave to propagate through the conductive medium, biological tissue in this case, at approximately 1500 m s^−1^ and can be detected as acoustic waves [6]. These acoustic waves can be used to map the optical absorption of the target. Depending on the image formation methods, PAT can operate as photoacoustic microscopy (PAM), or photoacoustic computed tomography (PACT) [3]. PAM raster scans a focused ultrasonic transducer across the imaging target and collects the resulting acoustic signals, while PACT produces images via large-area illumination of the object and parallel signal collection via ultrasonic transducer array and inverse reconstruction algorithms [2, 7]. Figure 1 illustrates the basic principle of PAT.

**Figure 2:**
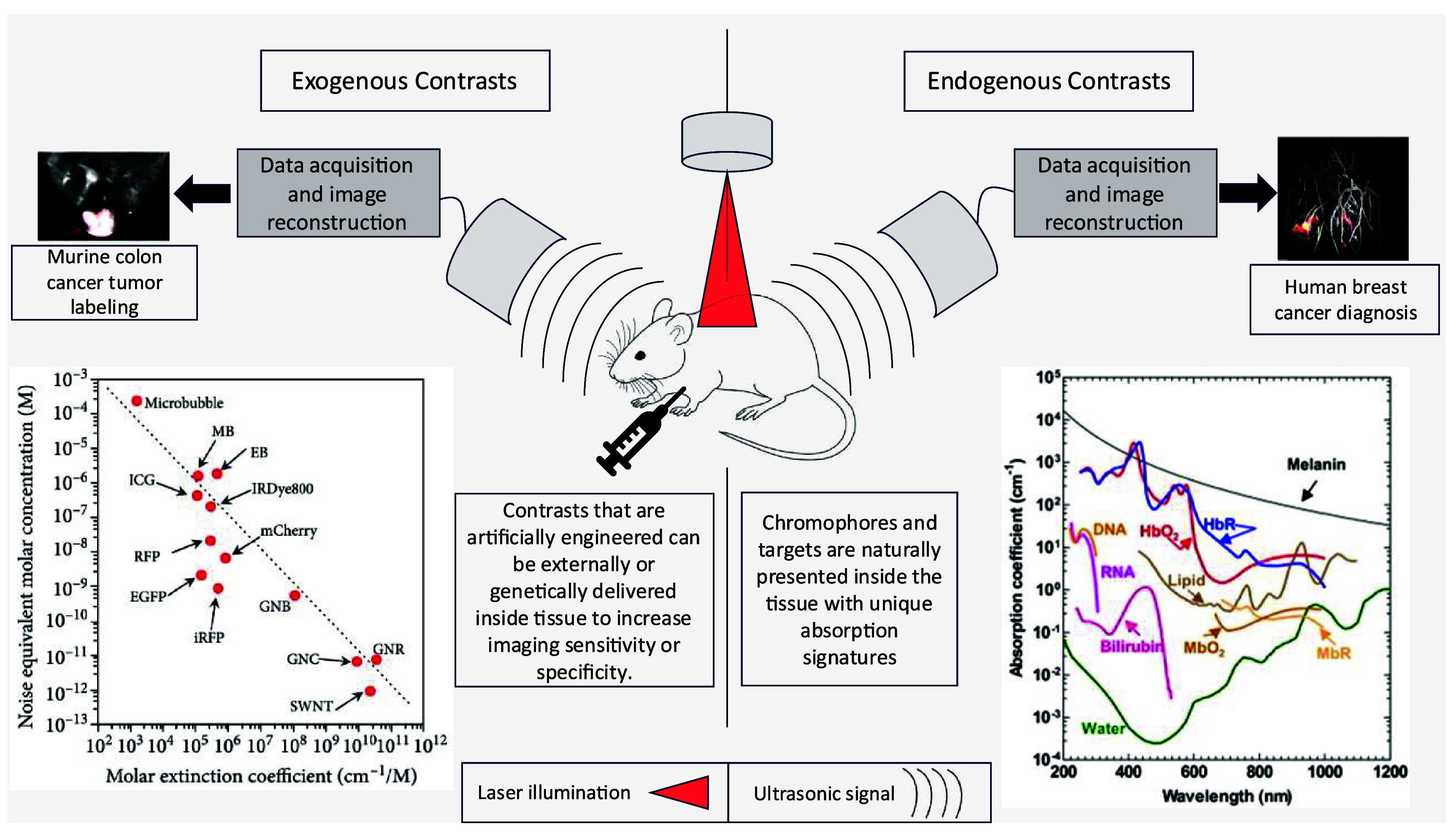
Article structure outline. Human breast cancer diagnosis image. Reproduced from [35]. CC BY 4.0. Endogenous contrast absorption spectra graph. Reproduced from [2]. CC BY 4.0. Murine colon cancer tumor labeling image. Reproduced from [36]. CC BY 4.0. Exogenous contrast absorption spectra graph. Reproduced from [2]. CC BY 4.0.

Due to the ability of PAT to use the optical absorption spectral signature of molecules, it can selectively image molecules by tuning the excitation wavelength to each target’s peak of optical absorption[8]. Biological systems rely on numerous endogenous molecules to carry out metabolic processes, all of which are potential imaging targets for PAT. The endogenous contrast agents include hemoglobin, myoglobin, melanin, lipids, nucleic acids, and glucose, among others [9–22]. However, there are applications where improved sensitivity and specificity are desired beyond what endogenous agents can provide. In these instances, exogenous contrasts, including genetically encoded proteins, organic dyes, and micro/nanoparticles, offer improved imaging capabilities in deep tissue [23–28]. Figure 2 shows an overview of select endogenous and exogenous contrast agents, their absorptions at varying concentrations and light wavelengths, and example applications for each type of contrast modality.

PAT possesses high spatial resolution at much greater imaging depth than pure optical systems by converting photons into acoustic waves, which undergo orders of magnitude less scattering inside tissue than photons [2, 29]. Using phosphorus phthalocyanine dyes at 1064 nm, PAT has achieved a maximum imaging depth of 11.6 cm [30],highlighting the greatly improved depth capability of PAT over optical imaging. Resolution in PAT systems can be improved by increasing the detection frequency of US transducers within the system while compromising the penetration depth [31].

In this review, we highlight a subset of PAT research that focuses on various molecular imaging techniques for visualizing structures, functions, and dynamics, utilizing both endogenous and exogenous contrasts, as summarized in table 1. Articles were chosen based on date of publication being within the last 10 years, with an emphasis on publications from well-respected, high-impact journals, and included those with a focus on imaging molecular targets *in vivo*, which can be used to evaluate metabolic processes and structures.

**Table 1:** Comparison of various contrast agents.

	Imaging depth	Imaging wavelengths	Specificity	Categories	Applications
Endogenous contrast agents	Up to 7 cm [34]	532–558 nm, 830–835 nm, 1064 nm	Moderate	Hemoglobin	Bone healing, breast cancer monitoring, arteriole and venule imaging
		600–680 nm	High	Melanin	Melanoma detection
		700–970 nm, 1730 nm	Moderate	Lipids	Human carotid artery lipid content assessment, Hepatic Steatosis evaluation
		266 nm	High	Nucleic acids	Osteoblastic Osteosarcoma microscopy, murine hippocampus microscopy
		3508 nm (2850 cm^−1^)	Poor	Glucose	Blood glucose concentration

Exogenous contrast agents	Up to 11.6 cm[30]	488 nm, 600–680 nm, 635–750 nm	High	Proteins	Genetically encoded indicators, photo-switchable phytochromes, calcium concentration indicators
		1064 nm, 800–1350 nm	High	Organic dyes	Photothermal therapy, reactive species, enzymes
		532–590 nm, 1000–1100 nm	Moderate to High	Nanoparticles	Blood clots, choroidal neovascularization

Molecular PAT holds immense potential for medical imaging due to its ability to visualize the structure and function of biological systems with high sensitivity and specificity [2, 32–34]. In this review, we have selected examples of a variety of clinical and preclinical research in molecular PAT to highlight its effectiveness in studying biological structures, metabolic processes, organ functions, and disease progression within living systems. To this end, we focus on the development and utilization of molecular contrast agents *in vivo* while providing only basic technical specifications of the associated imaging systems to give readers necessary context.

## 2 Endogenous contrasts

Naturally occurring biomolecules are of great interest to use in photoacoustic imaging due to their biocompatibility, usefulness as biomarkers in monitoring metabolic processes, and the ability to perform noninvasive imaging while targeting these molecules. The main sources of endogenous contrast include lipids, glucose, hemoglobin, nucleic acids, and melanin. Each of these molecules provides unique advantages that can be leveraged to image the desired processes and structures.

### 2.1 Lipids

Lipid quantification within the body targets several diverse areas of clinical significance. Both intravascular and extravascular imaging of the lipid contents in atherosclerotic plaques provide valuable information in assessing the age of the lesions as well as their likelihood of rupturing and causing an acute infarction in the tissue downstream of the occlusion [11, 37–40]. Additionally, quantification of lipid content is valuable in assessing hepatic steatosis [41–44], breast cancer tumor size [45, 46] and composition [9, 47], imaging neural tissue and kidneys [48],as well as tissue trauma-induced fat necrosis [49].

**Figure 3:**
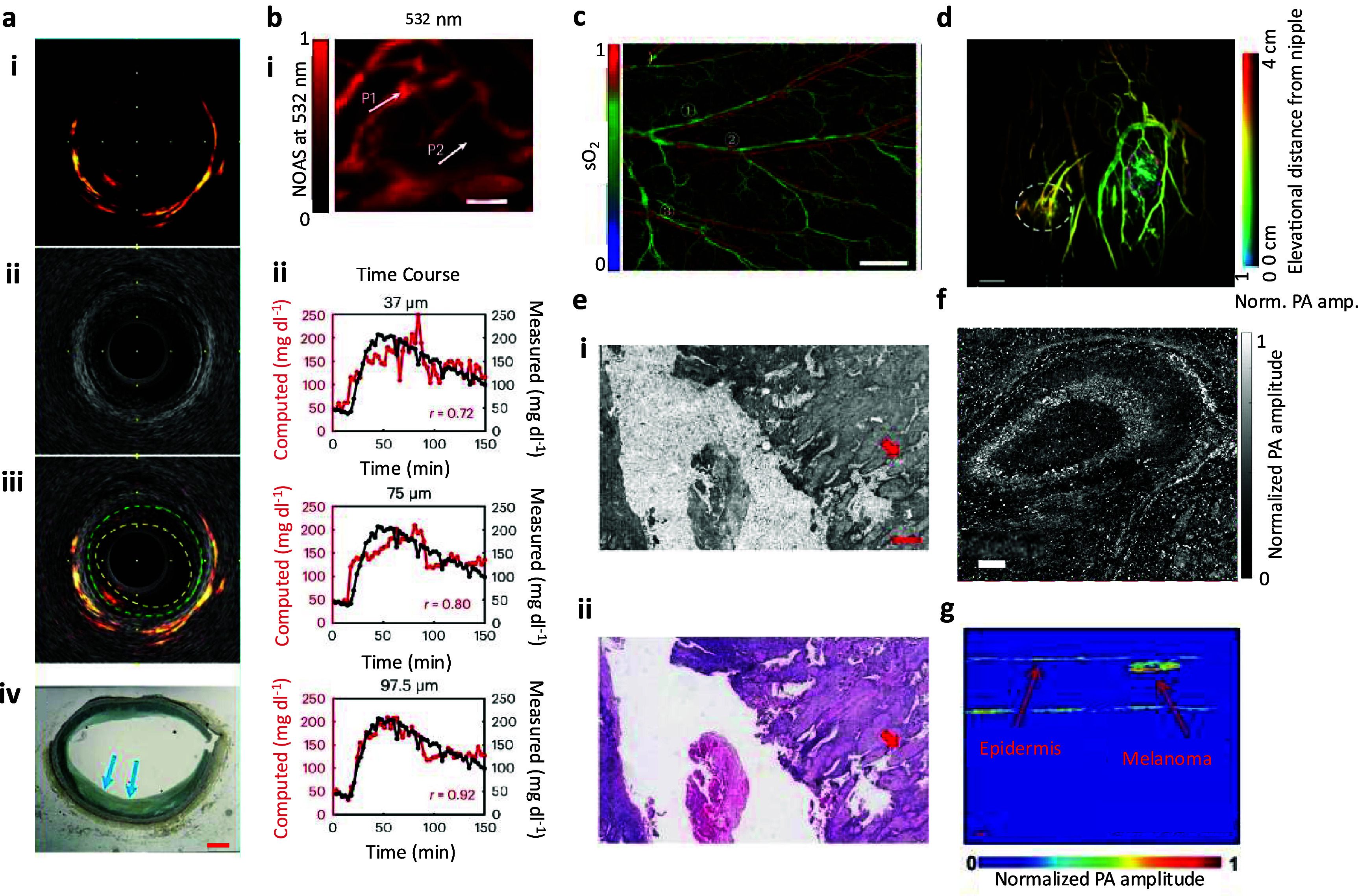
(a) *Ex vivo* IVPA imaging of a human right coronary artery at 1730 nm. (i) Representative cross-sectional PA images, (ii) US images, (iii) merged PA/US images and (iv) their corresponding Movat’s pentachrome-stained histopathology sections. Scale bar is 500  *μ*m. Reproduced from [11]. CC BY 4.0. (b) (i) Photoacoustic micrograph (532 nm wavelength), representative of ten independent experiments (*n* = 10). (ii) Time course of glucose measurements at different depths (37, 75 and 97.5 *μ*m) at P1, compared with reference blood glucose values when faced with glucose challenge test. Scale bar is 100 *μ*m. Reproduced from [13]. CC BY 4.0. (c) *In vivo* Optical Resolution OR-PAM of oxygen saturation in a mouse ear with ultrafast dual-wavelength excitation at wavelengths of 532 and 558 nm in pulse separated by 50 ns. (C) Oxygen saturation image of the mouse ear calculated from separate 532 and 558 nm pulses. The artery–vein pairs are labeled as 1, 2, and 3. Scale bar is 800 *μ*m [53]. John Wiley & Sons. © 2020 WILEY-VCH Verlag GmbH & Co. KGaA, Weinheim. (d) SBH-PACT of a cancerous breast. Depth-encoded angiogram of the affected breast with tumor identified by a white circle. Nipple marked with a magenta circle. Patient is a 48 year-old female with an invasive lobular carcinoma (grade 1/3). Scale bar is 1 cm. Reproduced from [35]. CC BY 4.0. (e) (i) UV-PAM image of undecalcified patient bone sections on a glass slide from a patient with osteoblastic osteosarcoma showing neoplastic osteoid matrix, denoted by red arrows. (ii) Corresponding H&E image acquired by a digital whole-slide scanning microscope with a ×40 objective, with an essentially identical appearance. Scale bars, 500 *µ*m. Adapted from [12], with permission from Springer Nature. (f) UV-PAM MAP image of the PA signal of a fixed mouse’s brain slice; close-up image of the hippocampal region. Scale bar is 200 *μ*m. Copyright [20] (2018) Society of Photo Optical Instrumentation Engineers (SPIE). (g) Melanoma image acquired with PAT, clearly showing the melanoma and skin surface with a photoacoustic depth of 1.9 mm. Scale bar is 1 mm. Reprinted from [54], Copyright (2017), with permission from Elsevier.

An Intravascular photoacoustic tomography (IVPA) system was developed using a 45° gold coated optical fiber end mirror paired with a 0.5 × 0.6 × 0.2 mm^3^, 42 MHz, 50% bandwidth US transducer and an OPO laser emitting 10 ns pulses at 1730 nm and 2 kHz repetition rate [11]. The lateral resolution was found to be 170–450 *µ*m with increasing depth while the axial resolution ranged from 85 to 100 *µ*m. Using a laser fluence of 50 mJ cm^−2^, rabbit aortas were first imaged *in vivo. Ex vivo* human carotid arteries were then assessed for lipid content and compared to Movat’s pentachrome-stained histology sections of the artery. Figure 3(a) depicts one of the human *ex vivo* carotid arteries imaged with photoacoustic (PA), US, and Movat’s pentachrome histology staining. Results of the study indicated excellent overlap in PA signal and areas of lipid deposition within the carotid artery histology slides.

PA evaluation of hepatic steatosis at bedside provides an interesting prospect for increasing the amount of screening for this disorder. Current methods utilize low throughput and expensive MRI [50, 51] or liver biopsy [52] which carries risks associated with any invasive procedure. A handheld system comprising a 256-element transducer array with 4 MHz central frequency in a 145° arc paired with a 10 ns pulsed laser output wavelength ranging from 700 nm to 970 nm in 10 nm increments and 3.75 mJ cm^−2^ fluence was used to evaluate hepatic steatosis in five steatosis patients [41]. With a spatial resolution of ∼100–300 *µ*m, the group was able to differentiate the relative lipid concentrations in the liver among hepatic steatosis patients compared to healthy control and coregister the images on US B-scans [41].

### 2.2 Glucose

Glucose concentration measurements within the bloodstream are critical for the management and treatment of diabetes [55]. Most current devices use invasive finger pricks to collect blood samples, which are then analyzed to determine glucose concentration in the blood. Photoacoustic quantification of blood glucose provides another, less traumatic avenue for monitoring blood glucose [13, 56–59].

Glucose and water share very similar light absorption spectrums, necessitating advanced time-delay filtering algorithms to discern glucose concentration from background water absorption using PA means [13]. Figure 3(b) shows a depth-encoded blood glucose measurement using PAM at 2,850 cm^−1^ compared to invasive glucose measurements. The position of the glucose measurements was aligned with hemoglobin imaging at 532 nm to ensure the precise location of the superficial murine ear microvasculature *in vivo* [13]. Images were acquired using a pulsed quantum cascade laser capable of emitting 3.4 *µ*m–11 *µ*m light with 20 ns pulses at a repetition rate of 100 kHz. For hemoglobin imaging, a 3 ns pulsed 532 nm laser was used. A 21 MHz central frequency US transducer was used for acoustic signal detection. The axial resolution was found to be 30 *µ*m.

### 2.3 Hemoglobin

The most widely used endogenous agent in PAT is hemoglobin. PAT can image both oxygenated and deoxygenated hemoglobin due to their unique light absorption spectra [60]. By altering the wavelength of illumination light, the concentration of oxy- and deoxy-hemoglobin can be determined at both the macroscopic [38, 61, 62] and microscopic levels [53, 61, 63, 64]. Hemoglobin is abundant in blood, making it an ideal endogenous contrast for vascular imaging as well [38, 53, 61–63, 65–67]. Applications of imaging vasculature are highly varied, including imaging of microvascular hemodynamics [53, 61, 68], tracking bone healing [63], identifying cancerous tumors [35, 69, 70], measurement of core body temperature [71],detection of anemia [72],monitoring of chronic foot ulcers [73] and vascular dysfunction [74], and determination of atherosclerotic plaque status in conjunction with lipid content [75].

Using a Vevo LAZR system with a 256-element linear-array transducer at 40 MHz center frequency and 750 nm as well as 850 nm laser illumination, the progress of bone healing was monitored [63]. Cd-1 mice were sacrificed for image collection at 2, 5, and 10 weeks post-surgical femur fracture, where conventional x-ray, micro-CT, and PA imaging compared union and non-union fracture repair progress. Union fracture repairs exhibited increased concentrations of hemoglobin and oxygen saturation (sO_2_) in callus tissue repairing the bone compared to non-union repairs, highlighting the importance of blood flow in bone healing [63].

An example of sO_2_ measurements performed using OR-PAM is shown in figure 3(c). Using a 532 nm 7 ns pulsed laser, 10 m polarization-maintaining single-mode optical fiber to provide 50 ns time delay and redshift the secondary laser pulse to 558 nm, and 50 MHz center frequency delay line transducer, dual-wavelength images were created [53]. With a lateral resolution of 3.6 *µ*m and axial resolution of ∼45 *μ*m, arteriole and venule pairs were able to be imaged and color-coded according to oxygen saturation in a murine ear. Mapping of oxygen saturation is critical for detecting regions of reduced blood flow which can lead to tissue necrosis and dysfunction if blood flow is not restored in a timely manner.

Elsewhere, Single breath-hold SBH-PACT has been developed for the detection of breast cancer without ionizing radiation as in traditional mammograms [35]. Using a 1064 nm, 10 Hz, 8–12 ns pulse width laser delivering 20 mJ cm^−2^ to the skin surface paired with a 512-element (2.25 MHz center frequency, >95% one-way bandwidth) full ring ultrasonic array possessing a spatial resolution of 255 *µ*m in-plane and elevational resolution of 5.6 mm, cancerous tumors of the breast were imaged [35]. Figure 3(d) shows a depth-encoded map of one patient with a grade 1/3 invasive lobular carcinoma highlighted by a white circle. Angiogenesis inside aggressive breast tumors, with abnormally high hemoglobin concentration, is the hallmark of differentiating lesions from normal breast tissue.

### 2.4 Nucleic acids

Nucleic acid imaging exists mainly as a method for microscopic imaging and relies heavily on ultraviolet (UV) wavelengths of light where DNA and RNA exhibit peak absorption. Research focuses on tissue microscopy of the brain [20, 21, 76–79] and cancerous tumors [21, 77] along with kidney [80], heart [80], colon [81], liver [81], bone [12], and virtual histological staining [12, 21, 77–79, 81].

Figure 3(e) depicts the result of a recent study using UV-PAM to image osteoblastic osteosarcoma in an undecalcified bone segment compared to its corresponding decalcified H&E stained light microscopy image [12]. The image was acquired using an neodymium-doped yttrium lithium fluoride (Nd:) Q-switched 266 nm nanosecond laser paired with a ring-shaped 42 MHz center frequency, 76% −6 dB two-way bandwidth US transducer possessing a lateral resolution of 0.96 *µ*m. The sample was prepared as a >1 cm thick rough bone segment fixed in formalin without further preparation and submerged in a water bath to ensure acoustic coupling. Of critical note for this methodology, rapid freeze-drying for traditional intraoperative H&E staining of the tumors is not possible due to the need for decalcification of the tissue before light microscopy and staining can be done. The total time for this process is on the order of days to one week [12]. The time for virtual H&E staining with the UV-PAM system was 11 min [12].

Highlighting the capability of UV-PAM to also image brain tissue, Imai *et al* utilized a 266 nm nanosecond laser with a 10 kHz repetition rate forming 40 optical foci, paired with a 256-element, 50 MHz central frequency focused 1-D US array, on imaging various areas of a murine brain [20]. The lateral resolution of the system was 1.6 ± 0.2 *μ*m with an axial resolution of 40.9 ± 6.9 *μ*m. Figure 3(f) shows a close-up image of a murine hippocampus. It was also found that individual nuclei around 5 *µ*m in diameter were also able to be imaged. Multifocal UV-PAM allows for intraoperative tumor sectioning to determine surgical margins and potentially prevent repeat procedures from removing remaining cancerous tissue.

### 2.5 Melanin

Melanin exists as a uniquely versatile biomolecule as it is fairly rare within the body outside of the skin, pigmented layer of the retina, hair, and areas of the brain and adrenal glands. Much research involving melanin revolves around imaging and quantifying melanomas [54, 82–87], melanoma metastases [10],and melanoma treatment response [88].

Figure 3(g) shows one such example of a melanoma imaged using a Vevo LAZR system equipped with a 256-element linear array, center frequency of 21 MHz, 70% one-way bandwidth, lateral and axial resolution of 119 *μ*m and 86 *μ*m, and a 680 nm illumination wavelength [54]. The results indicated a strong correlation (R^2^: 0.97) with actual Breslow’s depth as determined by surgical excision of the tumor after imaging was complete. Breslow’s depth is a crucial measurement for the determination of surgical resection margins so as to ensure the whole melanoma is removed without the need for subsequent surgeries [89].

Melanin is also useful as a source of contrast within exogenous agents such as gold nanocages or genetically encoded indicators. These will be addressed later in this review.

Advantages of endogenous contrast agents include that they enable noninvasive imaging of structures and functions thanks to their natural biocompatibility, as well as the capability to monitor processes such as wound healing due to their innate and integral role in the same process [90]. The hemoglobin contrast is useful for this study as it is contained within the red blood cells responsible for delivering nutrients needed for regeneration and normal tissue function. However, due to its high concentration within blood and wide distribution throughout the body, it lacks the specificity desired for many clinical and basic science research investigations. Naturally occurring hemoglobin, lipids, nucleic acids, and glucose are all found in both healthy and diseased tissue, limiting their ability to distinguish lesions from normal tissue, especially in the early stage [91, 92]. Moreover, the low optical absorption of lipids, proteins, and glucose within the near-infrared (NIR) wavelength region also restrains the imaging sensitivity [2, 93]. Thus, the limited specificity and sensitivity of endogenous contrast stimulate the development of exogenous contrasts [2].

## 3 Exogenous contrast agents

Endogenous contrast agents are sometimes inadequate for PAT applications for several reasons. They are normally ubiquitous within a region of interest, contributing to background signals during imaging, which can completely overwhelm the desired targets. To overcome this, researchers have developed exogenous contrast agents working in the NIR region. NIR light has minimal optical attenuation in biological tissues, allowing for deeper tissue penetration. Moreover, the American National Standards Institute permits higher energy levels for NIR wavelengths, further enhancing imaging depth and signals in deep tissue.

Specifically, imaging in the NIR-II window (1000–1700 nm) offers distinct advantages over the NIR-I window (700–1000 nm). NIR-II imaging allows for deeper tissue penetration due to higher maximum permissible exposures (MPEs). For instance, at 800 nm, the MPE is approximately 300 mW cm^−2^ per laser pulse, whereas at 1064 nm, it increases to around 1000 mW cm^−2^.

Endogenous agents are also not able to selectively target certain structures or molecules, such as cell surface markers, necessitating agents with improved specificity. Finally, exogenous agents also allow for functional imaging applications where specific biological processes cannot be visualized using endogenous contrasts. Three major types of exogenous contrasts, including genetically encoded proteins, organic dyes, and nanoparticles, will be reviewed.

### 3.1 Proteins

Genetically encoded proteins hold several unique advantages for PAT. They are highly customizable, enabling researchers to obtain a wide variety of desirable contrast characteristics [94]. For example, photoswitchable phytochromes can change their optical absorption at specific wavelengths upon exposure to an activating excitation, showing unique optical properties different than any other endogenous chromophores [23, 95–101]. Other proteins can also be used to target specific biological processes through selective molecule binding, such as tracking neuronal calcium levels in the brain, where increased calcium concentrations induce conformational changes in the protein indicators and increase their absorption coefficients during nerve activation [102–106]. Finally, selectively labeling cells for detection of gene expression and cell growth over time using genetically encoded markers is also possible with enzyme based modalities, such as tyrosinase (Tyr), that produce photoactive proteins like eumelanin [36, 107–110].

**Figure 4:**
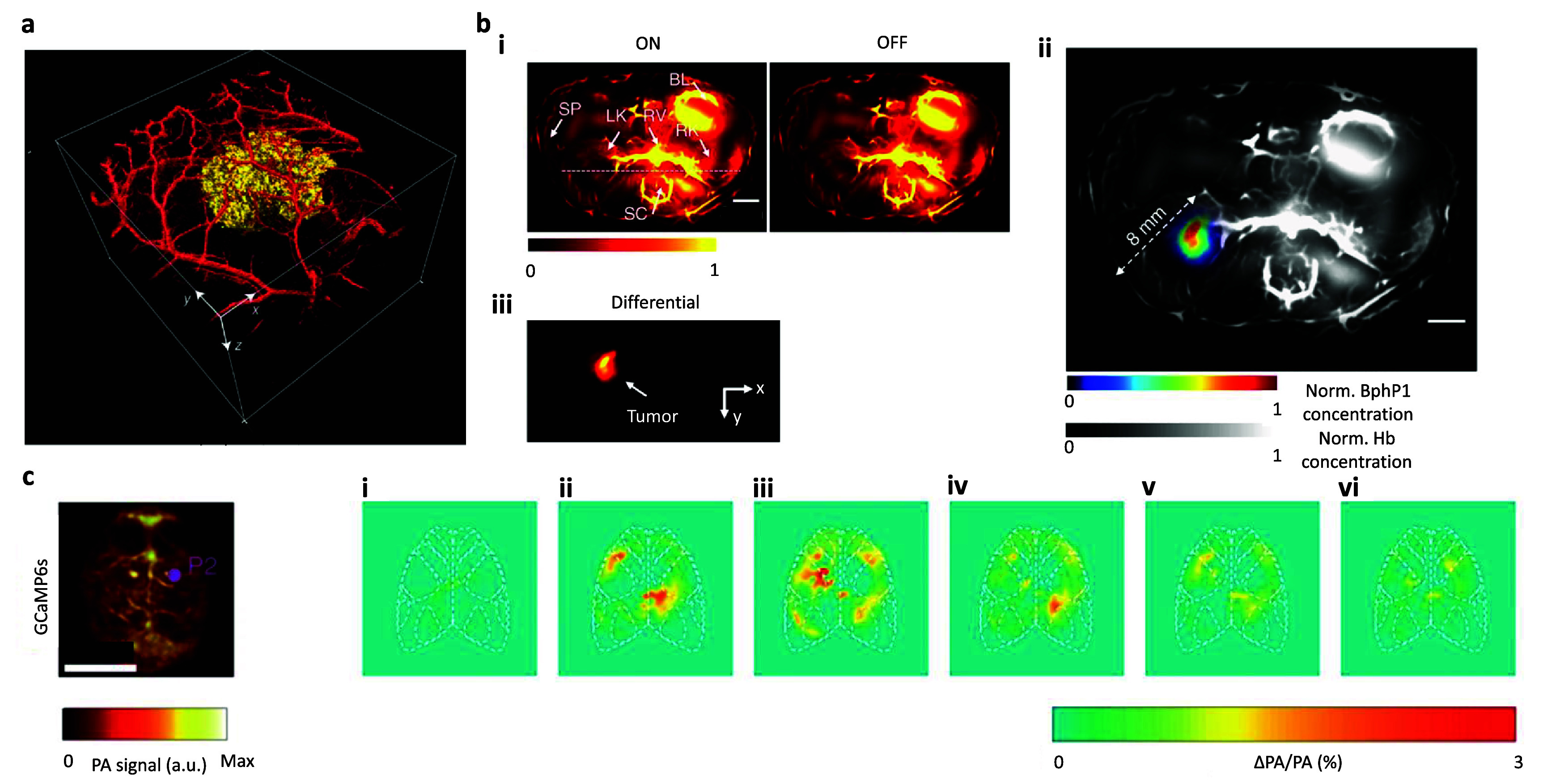
(a) *In vivo* PA images of Tyr-expressing K562 cells after subcutaneous injection into the flank of a nude mouse (*λ*_ex_ = 600 nm). Volume-rendered image (day 0). Section is 14 mm × 14 mm × 6 mm. Adapted from [108], with permission from springer nature. (b) The PA images were acquired at 750 nm. (i) *In vivo* whole-body PACT images of the kidney region of a nude mouse, acquired 1 week after injection of BphP1-expressing U87 cells into the left kidney. The ON- and OFF-state PA images show the major blood-enriched internal organs, including the left kidney (LK), right kidney (RK), spinal cord (SC), renal vein (RV), bladder (BL) and spleen (SP). (ii) An overlay of the U87 tumor (in color) in the left kidney and the blood-dominated OFF-state image (in grayscale). Hb, hemoglobin. (iii) The differential image showing the left kidney. The scale bar is 3 mm in all images. Adapted from [96], with permission from springer nature. (c) Depiction of a GCaMP6s expressing mouse response to electrical stimulation of the left hind paw maximum intensity projection along the depth direction of the 3D image. Relative increase in OA signal with respect to the baseline for a slice at approximately 1 mm depth at different time points following the stimulation pulse for a GCaMP6s-expressing mouse. Frames depict the signal from left to right at (i) 0 ms, (ii) 240 ms, (iii) 360 ms, (iv) 480 ms, (v) 720 ms, (vi) 960 ms. Scale bar is 5 mm. Adapted from [103], with permission from Springer Nature.

In an effort to increase specificity during PA imaging, a team recently developed a Tyr expression-based genetically encoded reporter for tracking individual cells [108]. This technique introduces the Tyr enzyme to the cell’s phenotype, which begins producing the pigment eumelanin within the cytoplasm of cell [108]. The experiment used an all-optical US detector based on a high finesse FP polymer film etalon with a broadband frequency response ranging from 350 KHz to 22 MHz (−3 dB). This was paired with 50 Hz laser pulses varying between 600–680 nm at 1.5–1.7 mJ cm^−2^ fluence, depending on the exact experiment being performed, yielding a spatial resolution of 100 *µ*m at the center of the image to 150 *µ*m at its extremities at a depth of 8 mm. Retroviral transfection of 293 T cells was undertaken to introduce a Tyr-expressing gene into the genome of the cell line, allowing for the identification of both the host cell and its subsequent generations as the gene was passed down with each cell division [108]. These transfected cells were then observed as they multiplied and grew, with the newly produced eumelanin from the Tyr gene highlighting the cells and tumor of interest. Figure 4(a) shows a K562 and 293 T Tyr-expressing cell tumor implanted in the flank of a nude mouse, demonstrating the excellent contrast between the tumor and surrounding vasculature.

Elsewhere, photoswitchable phytochromes show great promise in increasing imaging sensitivity and specificity as they allow for significant suppression of background signals. Photoswitching involves molecules which typically express two conformational states which are induced by photoisomerization at molecule specific wavelengths of light [96]. In the ON state these molecules absorb light at longer wavelengths, such as the 730–790 nm range for BphP1, while in the OFF state they absorb more strongly at shorter wavelengths (630–690 nm for BphP1). Generally, incident light of one wavelength is used to image the protein at the ON state while gradually changing its conformation and switching it to the OFF state. Then, the activation light is used to switch the protein back to the ON state. This process can be repeated in order to demarcate the desired photoswitching signal from a background with an improved contrast-to-noise ratio. Figure 4(b) shows an example of this technique using a full ring transducer array. The ring is comprised of 512, 5 MHz central frequency trasnducers. This was paired with a 630 nm continuous wave laser diode and 780 nm pulsed light during the reversible photoswitching part of the experiment which produced radial and tangential resolutions of 100 *μ*m and 100–250 *μ*m, with the tangential resolution degrading with increased distance from the central focus point [96]. The protein used during imaging was transgenic Bphp1 expressed in U87 tumor cells implanted in the left kidney of nude mice. Using 16 s of 630 nm continuous light, the Bphp1 protein was ‘activated’ which altered its absorption peak to 780 nm. 16 s of pulsed 780 nm light was then used to simultaneously image the implanted tumor and deactivate the protein back to its original conformation where its peak absorption was at ∼630 nm.

Another valuable advantage of protein-based imaging contrasts is their ability to bind specific molecules or even ions. Using mice expressing the calcium indicator protein GCaMP6f, a team was able to image concentrations of calcium in murine brains while mapping changes over time in response to various hindleg stimulations [102, 103]. Using an 8 cm diameter hemispherical ultrasonic array comprised of 512 elements (5 MHz center frequency, 100% detection bandwidth of −6 dB) and a 25 Hz repetition, 488 nm laser at a fluence of 3 mJ cm^−2^ producing a nearly isotropic spatial resolution of 150 *µ*m, the GCaMP protein was imaged *in vivo*. The team demonstrated noninvasive imaging of the mouse brain activities over time, as shown in figure 4(c) when the mouse’s hind paws were stimulated with an electrical signal.

### 3.2 Organic dyes

Organic dyes, particularly those absorbing in the NIR-II region, have shown promise for photothermal therapy (PTT) due to their tunable optical properties and biocompatibility. Additionally, activatable PA probes, which respond selectively to specific biomarkers or molecular events, have been developed to enhance diagnostic sensitivity and specificity. These probes, activated by reactive oxygen species or caspase-3 enzymes, offer improved target-to-background ratios and enable real-time monitoring of therapeutic efficacy, showing potential for more accurate and personalized cancer treatments.

#### 3.2.1 Enhancing PTT efficacy in the NIR-II region

The excellent biocompatibility, biodegradability, and tunable optical properties of organic dyes make them particularly suitable for PTT. In recent years, NIR-II absorbing organic dyes have been increasingly reported for use in PTT.

**Figure 5:**
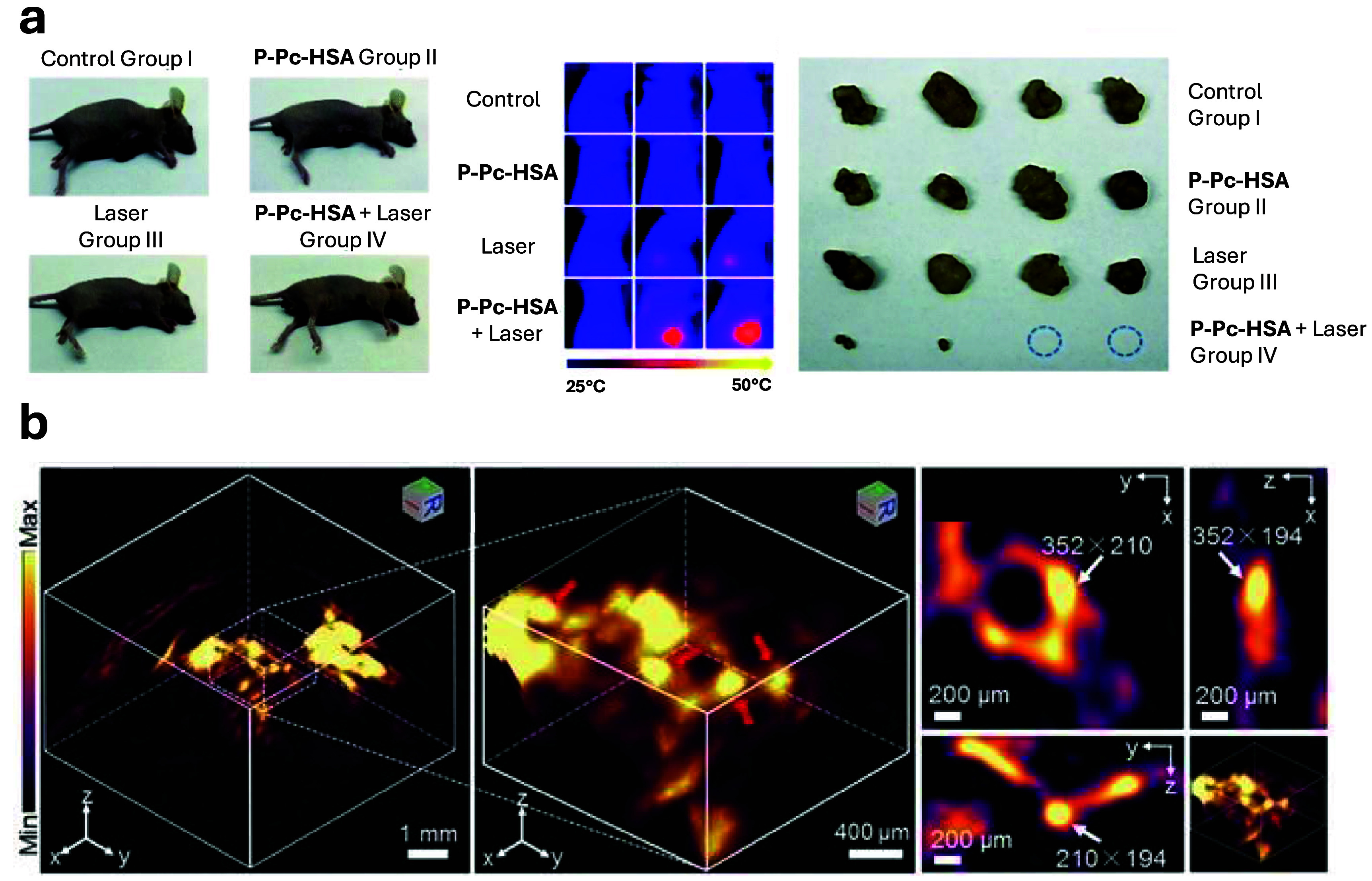
(a) (Left) Sixteen nude mice bearing tumors were randomly divided into four groups. Group I was the control group without any treatment. Group II was the dark group, and only P-Pc-HSA was administered (P-Pc-HSA, 50 ml, 144 mg ml^−1^, 8 min in dark). Group III was the laser group, and only light was administered (1064 nm laser, 1.2 W cm^−2^, 8 min). Group IV was the treatment group:4 h after the intratumor injection of P-Pc-HSA (the nanoagent still retained in the tumor principally at this time), 1064 nm laser was administered to perform PTT. (Middle) Thermal imaging on mice tumor shows the significant temperature increase. (Right) The rapid temperature increases at tumor sites resulted in an irreversible damage, and some tumors even disappeared. Reproduced from [113]. CC BY 3.0. (b) 3D Reconstruction PA image of apoptotic tumor (left) and 3D-slice in tumor tissues (right) in DOX-treated mice at 10 h after injection of 1-RGD. Red arrows showed the views of representative individual apoptotic region within the tumor tissues. White arrows show the same apoptotic region in the xy, xz, and yz panels [114]. John Wiley & Sons. © 2019 Wiley-VCH Verlag GmbH & Co. KGaA, Weinheim.

Phthalocyanines (Pcs) organic dyes have high optical absorption in the NIR and notable photothermal conversion efficiency, making them excellent candidates for PTT [111, 112]. However, one major limitation of Pcs is their poor water solubility, which has prevented their broader application in photothermal tumor treatment. To address this limitation, Zhou *et al* developed a molecular phosphorus phthalocyanine (P-Pc)-based nanoagent, which utilizes the high photothermal conversion efficiency of Pcs while improving biocompatibility through the use of human serum albumin (HSA) as a stabilizing agent. The use of HSA not only reduces toxicity but also improves water solubility, making the P-Pc nanoagent a promising candidate for clinical applications in cancer therapy [113]. Figure 5(a) shows the rapid temperature increases at tumor sites resulted in irreversible damage. This nanoagent demonstrated a remarkable photothermal conversion efficiency of up to 64.7% under 1064 nm light, showing its potential as a low-toxicity organic molecule-based agent for PTT cancer treatment.

**Figure 6:**
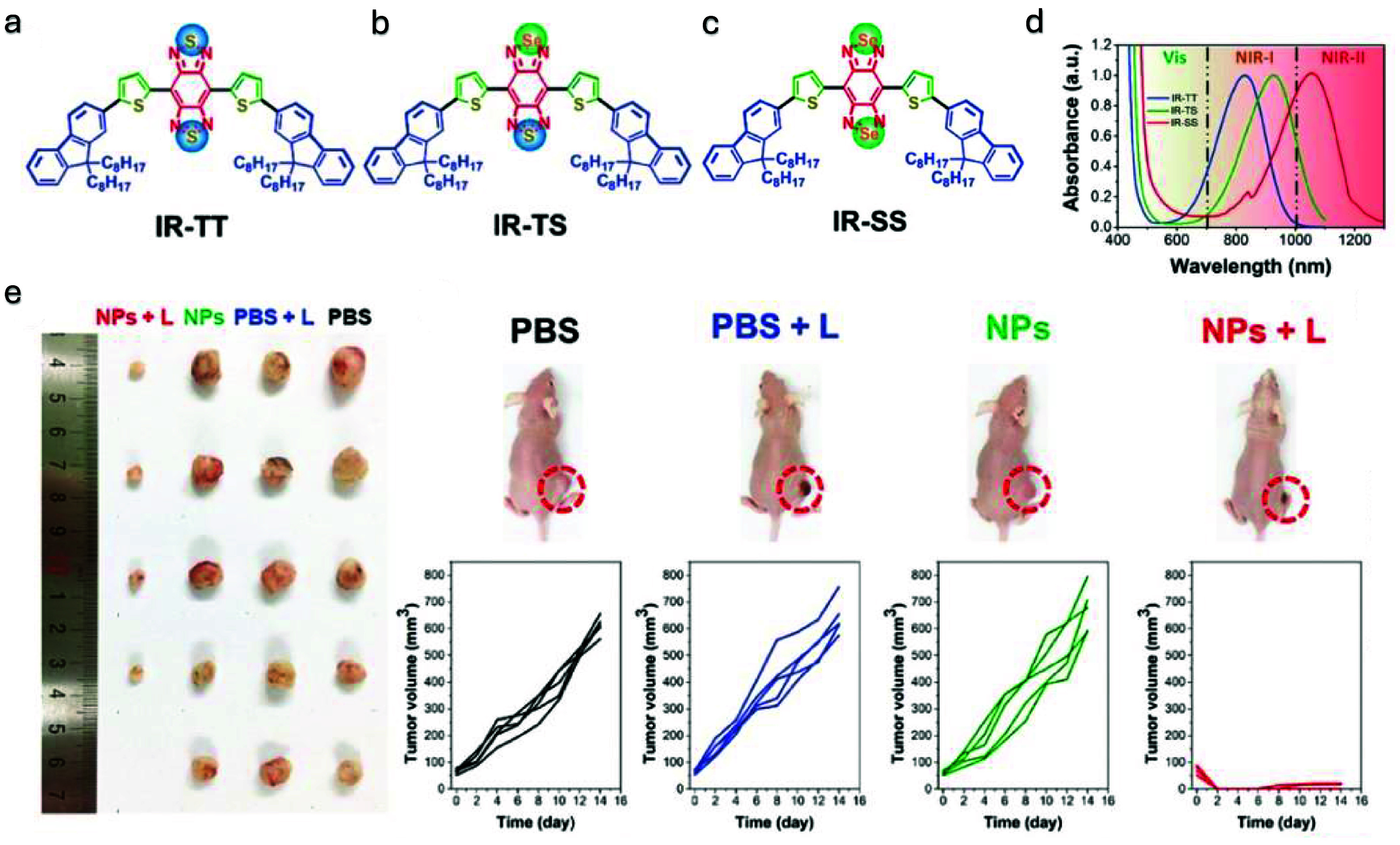
(a) IR-TT consists of D–π–A–π–D conjugated small molecules (as donor units) with thiophene serving as both the π-conjugation unit and the electronic bridge for ICT. A strong acceptor, benzo[1,2-c:4,5-c’]bis([1,2,5]thiadiazole) (BBT), is included to achieve a lower energy gap. To further redshift the absorption, sulfur (S) atoms in the BBT unit were replaced with selenium (Se) atoms. This led to the synthesis of IR-TS (b) and IR-SS (c), with one and two S atoms substituted, respectively. (d) The absorption peak of IR-TT is at 830 nm, while IR-TS and IR-SS exhibit shifts to 930 nm and 1060 nm, respectively [117]. (e) Tumor-bearing mice were uniformly classified as four groups (PBS, NPs only, laser only, and NPs + laser) after the tumor size reaches ≈80 mm^3^. the tumors display similar growth rates in the PBS, the laser only, and the NPs only groups. the mice in the NPs + laser group exhibited almost complete inhibition of tumor progress without any tumor recurrence in the next 14 d post-treatment. (a)–(e) [117] John Wiley & Sons. © 2020 WILEY-VCH Verlag GmbH & Co. KGaA, Weinheim.

In recent years, donor-acceptor (D-A) conjugated small molecules (SMs) have demonstrated exceptional light-harvesting and amplification capabilities, making them valuable in a range of biomedical applications. These D–A conjugated SMs are constructed from electron-rich donor units and electron-deficient acceptor units, resulting in efficient intramolecular charge transfer (ICT) due to the strong interaction between the donor and acceptor components [115]. This ICT endows the molecules with excellent light absorption properties and high extinction coefficients, making them effective light absorbers [116]. However, a key limitation in their current use is that most applications are confined to the visible spectrum, which restricts their effectiveness for deep tissue imaging. To address this limitation, Li *et al* developed a strategy to shift D–A SMs into the NIR-II region by substituting specific atoms (as shown in figure 6) [117]. By introducing a strong acceptor unit called benzo[1,2-c:4,5-c’]bis([1,2,5]thiadiazole), they created a molecule called IR-TT, which significantly enhances the absorption peak in NIR-I. To further shift the absorption peak from NIR-I to the deeper NIR-II region, sulfur (S) atoms were replaced with heavier selenium (Se) atoms, resulting in the molecule IR-SS. The IR-SS molecule is synthesized and exhibits a high photothermal conversion efficiency of 77%. As shown in figure 6(e), with intravenous injection and subsequent 1064 nm laser irradiation, IR-SS molecule caused a rapid increase in tumor temperature, leading to nearly complete tumor inhibition and significant apoptosis. This represents a crucial advancement in NIR-II nanomedicine, as it showcases the potential of SM-based photothermal agents for efficient and targeted cancer therapy, particularly in deep tissue applications [117].

#### 3.2.2 Activatable probes for enhanced specificity and sensitivity

Recent studies have highlighted activatable PA probes as a promising technology for enhancing diagnostic specificity and sensitivity. Unlike traditional ‘always-on’ probes, which emit signals regardless of target binding, activatable PA probes respond only to specific biomarkers or molecular events [118–121]. This selective activation significantly improves both the target-to-background ratio and the limit of detection [122], making them especially valuable for early disease diagnosis.

Activatable organic dyes are typically triggered by reactive species and enzymes. Reactive oxygen species (ROS) are a subset of reactive species, and oxidative stress associated with ROS can lead to various diseases, including cancer and cardiovascular disease. ONOO^–^, or peroxynitrite, is a kind of reactive oxygen molecule generated by the reaction of endogenous nitric oxide. ONOO^–^ is closely related to tumor immunosuppression, which enhances the understanding of its pathological function [123, 124]. Zhang *et al* introduced a water-soluble small-molecule probe (CySO_3_CF_3_), which operates in two states: a ‘caged’ form with weak fluorescence and an ‘uncaged’ form activated by ONOO^–^. Upon exposure to ONOO^–^, CySO_3_CF_3_ converts to CySO_3_OH, resulting in red-shifted NIR absorption. This conversion boosts PA signals by 5.1-fold under 680 nm light compared to the caged state. Its water solubility allows for *in vivo* imaging in mice, showcasing its potential for real-time diagnostics in living organisms [125].

Enzymes, which serve as biomarkers for disease progression, are also key activators of PA probes. Caspase-3 is a type of cysteine protease that induces the apoptosis pathway. Detecting caspase-3 activity and apoptosis is important for early evaluation of therapeutic efficacy and for selecting antitumor drugs [126, 127]. Wang *et al* developed a caspase-3-activatable PA probe, 1-RGD, designed to be efficiently delivered and activated by caspase-3. This probe integrates 2-cyano-6-hydroxyquinoline, d-cysteine, a caspase-3-cleavable peptide (DEVD), a glutathione (GSH)-reducible disulfide bond, and the NIR dye indocyanine green (ICG). In apoptotic tumors, 1-RGD self-assembles into high-density ICG nanoparticles due to interactions with intracellular GSH and caspase-3. This aggregation leads to aggregation-caused quenching, which enhances the PA signals. The larger ICG aggregates also have prolonged retention in tumor tissue, enabling high-resolution 3D imaging of localized tumor regions and allowing for early monitoring of therapeutic responses. Figure 5(b) shows a 3D reconstruction PA image of apoptotic tumor and 3D-slice in tumor tissues in DOX-treated mice 10 h post-injection of 1-RGD. It shows potential in analyzing the spatial distribution of apoptosis signals and can facilitate early and real-time evaluation of tumor therapeutic efficacy [114].

These advancements in activatable PA probes offer great potential for improving diagnostic accuracy and monitoring treatment efficacy.

### 3.3 Nanoparticles

Nanoparticles offer enhanced targeting, controlled drug release, and improved imaging capabilities, addressing the limitations of traditional contrast agents and therapies. Nanoparticles integrate properties like strong NIR absorption, selective targeting, and controlled delivery of therapeutic agents such as nitric oxide, improving diagnosis and treatment of thrombotic diseases [128, 129]. Advances in nanoparticle surface chemistry have also enabled more precise targeting and imaging, particularly in challenging areas like retinal vessel imaging and tumor vasculature detection.

#### 3.3.1 Nanoparticles for PAT and special functionality

Nanoparticles offer enhanced functionalities compared to other exogenous contrast agents. Song *et al* developed NIR-II B@SP–C nanoparticles capable of targeting blood clots and controlling nitric oxide (NO) release [24] to dissolve occlusive thrombi in blood vessels. These nanoparticles were synthesized using a D-A approach and a Stille cross-coupling reaction to create the PTIIG homopolymer (dibromo thienoisoindigo monomer polymerized with hexamethylditin). To enable clot targeting, a peptide called Cys-Arg-Glu-Lys-Ala (CREKA) was conjugated to DSPE-PEG2000-Mal and incorporated into the nanoparticle structure.

NO is a key component, as it naturally prevents thrombosis in healthy blood vessels [130]. To make the nanoparticles function not only as a contrast agent but also as a thrombolytic drug, a temperature-sensitive NO donor, BNN6, was synthesized and doped into the PTIIG matrix. This allows precise control of NO release by toggling the NIR laser between ‘on’ and ‘off’ states.

**Figure 7:**
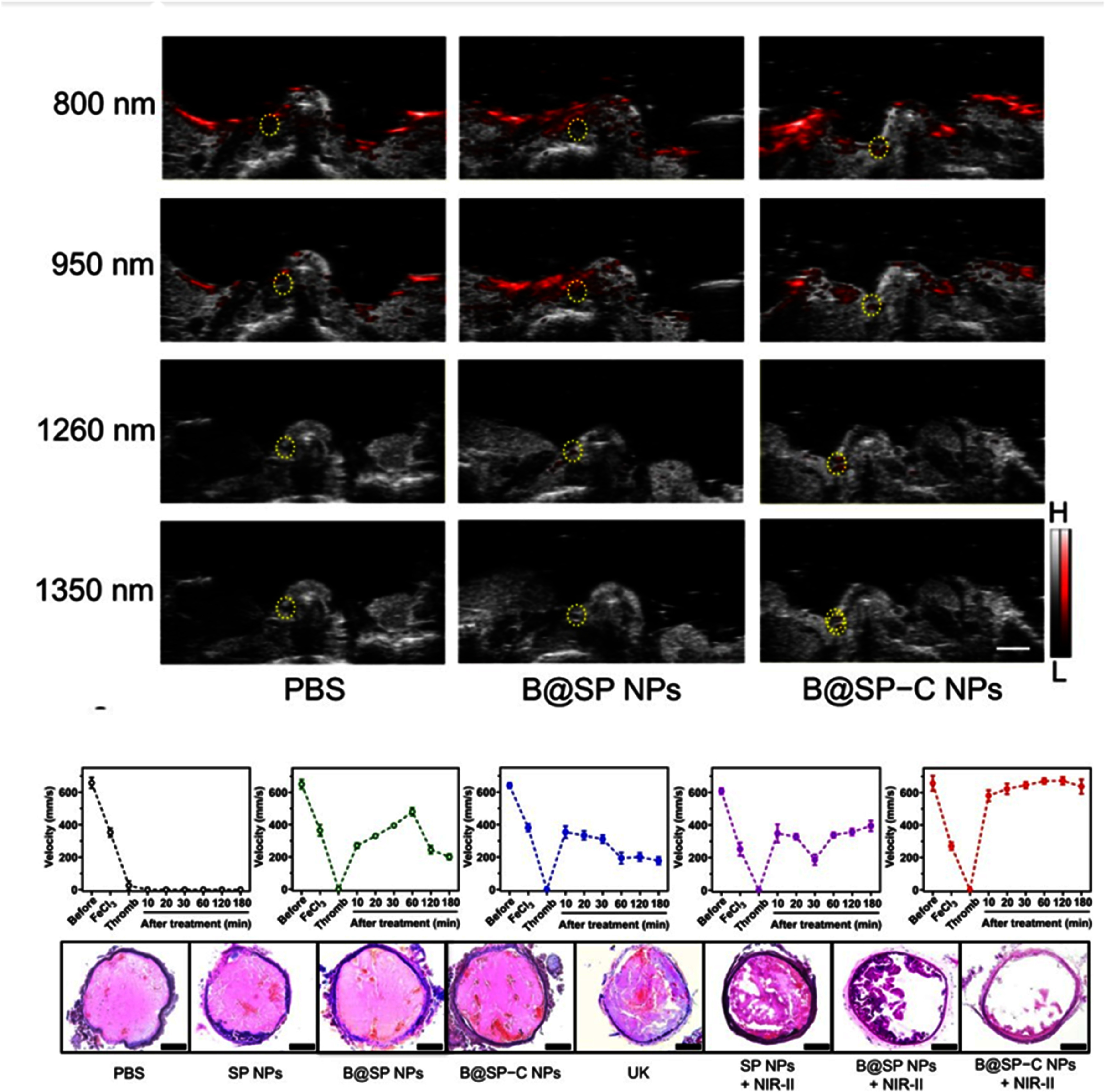
(a) Images of the blood clot under 800, 950, 1260, and 1350 nm, respectively. At 1260 nm, the nanoprobe exhibited a bright PA signal, while the background signal from endogenous absorbers was weak (top). After all treatments (PBS, SP NPs, B@SP NPs, B@SP-C NPs, free UK, SP NPs + NIR-II laser, B@SP NPs + NIR-II laser, and B@SP–C NPs + NIR-II laser), the carotid arteries were collected for sectioning. H&E staining of the vascular sections was then performed to visualize the embolism in the vessels. Quantitative data revealed that the relative volume of clots in the B@SP − C NPs + NIR-II group was reduced by approximately 90% (bottom). (a) Reproduced from [24]. CC BY 4.0.

For *in vivo* PA imaging, a 1260 nm excitation wavelength was selected, as the nanoprobe exhibited a bright PA signal while the background signal from endogenous absorbers remained weak. The PA imaging system was set with the following parameters: frequency = 30 MHz; wavelength range = 680–970 nm and 1200–2000 nm; imaging wavelengths = 800 nm, 950 nm, 1260 nm, and 1350 nm; and scanning area ∼165 mm^2^. Figure 7 shows a strong PA signal from the blood clot and a significant reduction in clot size in the B@SP − C NPs + NIR-II group after treatment.

The controlled delivery system in these nanoparticles addresses several limitations of current anti-thrombotic drugs. Clinically used drugs often have short circulation times, limiting their effectiveness in the bloodstream [130, 131]. Additionally, their therapeutic window is narrow, meaning they must be administered in precise dosages to avoid side effects. Their targeting efficiency is usually low, with less than 5% of the drug reaching the thrombus [132, 133]. Finally, these drugs face challenges in penetrating thrombi effectively. The nanoparticle-based system overcomes these issues by prolonging NO release at thrombotic sites, enhancing both targeting and penetration for better therapeutic outcomes.

#### 3.3.2 Gold nanoparticles for imaging retinal vessels

Advancements in nanoparticle surface chemistry have enabled precise control over their interactions with biological targets. Surface functionalization, in particular, allows for the creation of customized nanoparticles that selectively bind to specific molecules or cells, enhancing the sensitivity and specificity of PAT. By attaching proteins or aptamers to the surface of plasmonic nanoparticles (PNPs), their affinity for targeted molecules or cells can be significantly improved, thereby optimizing PAT performance [116].

Metal complexes offer additional advantages over organic PA probes due to their diverse NIR-absorbing properties, which can be finely tuned based on their metal centers, coordination geometries, and electronic configurations [134–137]. This flexibility makes them valuable for applications that are harder to achieve with organic probes. Nguyen *et al* developed chain-like gold nanoparticle (GNP) clusters conjugated with Arginylglycylaspartic acid (RGD) peptides (CGNP clusters-RGD) to label choroidal neovascularization (CNV) in living rabbits [138]. While colloidal GNPs are promising contrast agents for PAT, their absorption peak overlaps with hemoglobin, limiting their clinical use. To address this, Nguyen’s team synthesized GNPs with an average diameter of 20 nm using femtosecond pulsed laser ablation in deionized water. To form chain-like clusters, the GNPs were modified with ligands, including the pentapeptide Cys-Ala-Leu-Asn-Asn (CALNN) and cysteamine, through a sequential mixing process. These CGNP clusters were then conjugated with polyethylene glycol (PEG) and RGD ligands, resulting in clusters with an average length of 64 nm and excellent colloidal stability. The RGD-conjugated clusters specifically targeted *α*_v_*β*_3_ integrins, present in CNV but absent in normal vasculature, making them a promising diagnostic tool. For PAM imaging, a tunable nanosecond pulsed laser (405–2600 nm, 1 kHz repetition rate, 3–5 ns pulse duration) from a Q-switched Nd:YAG laser (NT-242, Ekspla) was used. The laser was collimated to a 2 mm circular spot and focused to ∼20 *µ*m on the fundus with an average pulse fluence of 0.01 mJ cm^−2^ at 578 and 650 nm. A 27 MHz needle transducer (−60% bandwidth, Optosonic Inc.) detected the PA signals, achieving lateral and axial resolutions of 4.1 *µ*m and 37.0 *µ*m, respectively.

**Figure 8:**
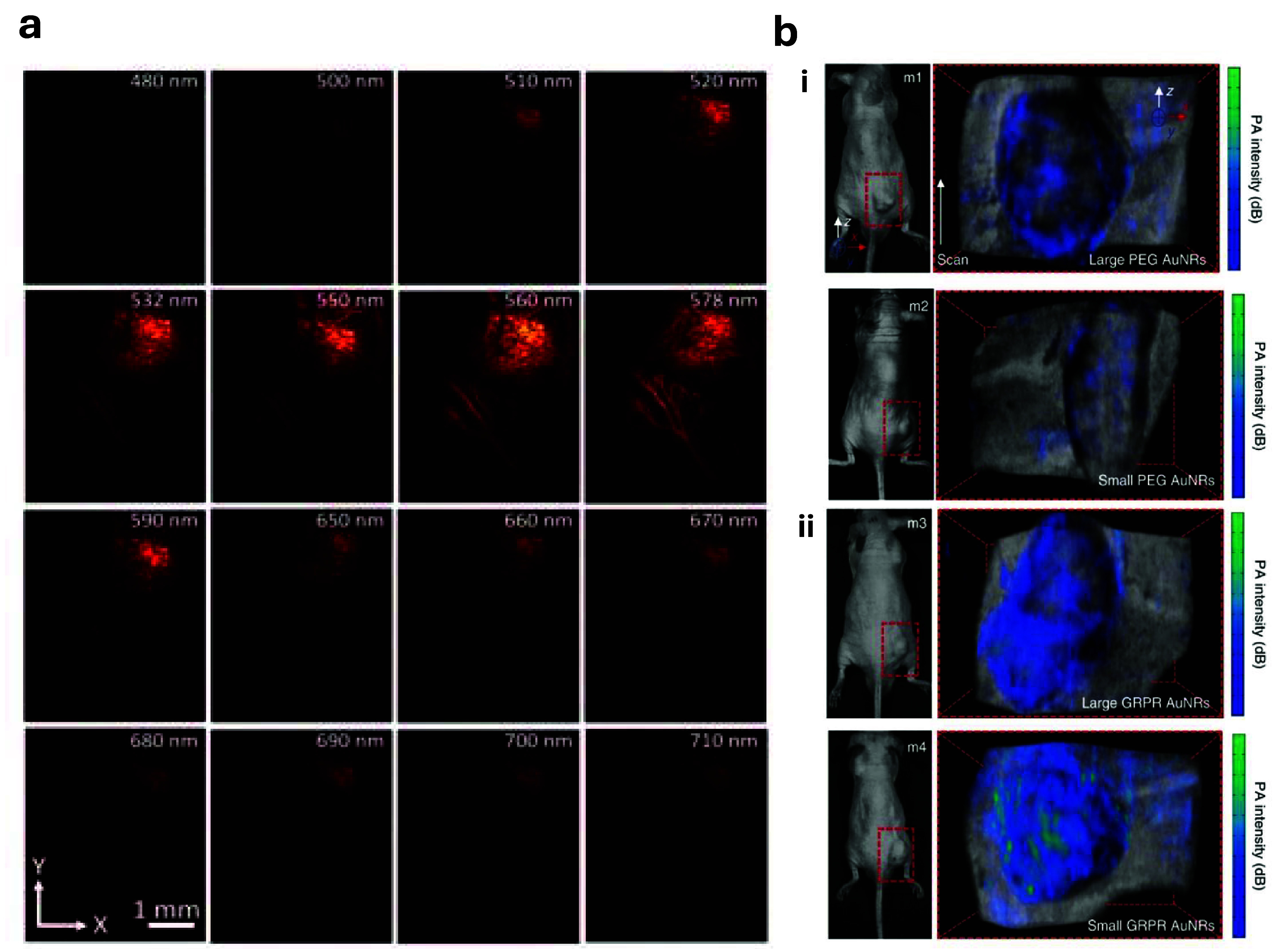
(a) PA signals at wavelengths ranging from 480 to 710 nm. At 650 nm, the best contrast was observed between healthy blood vessels and CNV. Reproduced from [138]. CC BY 4.0. (b) (i) Photographs and photoacoustic imaging of tumor-bearing mice (m1–m4) with non-targeted large and small AuNRs. (ii) Targeted large and small AuNRs. Adapted from [139], with permission from Springer Nature.

As shown in figure 8(a), at 578 nm, both healthy blood vessels and CNV are clearly visible. However, at 650 nm, the healthy blood vessel is completely absent, and only CNV can be seen, with its signal larger than at other wavelengths [138]. This study extended their application to rabbits, whose eye anatomy closely resembles that of humans, showcasing their potential for clinical use.

#### 3.3.3 Miniature gold nanorods with higher sensitivity

Metallic nanoparticles, particularly GNPs, exhibit favorable optical properties, including strong absorption in the red and NIR spectral ranges due to surface plasmon resonance. These PNPs typically achieve extinction coefficients exceeding 10^9^ M^−1^ cm^−1^, a level unattainable by small organic molecules. The absorption cross-sections of metal nanoparticles are expected to be an order of magnitude larger than those of organic dye molecules, leading to higher PA signals [140–142]. The absorption maxima and extinction coefficients of PNPs can be tuned by adjusting their shape, size, and material composition.

Gold nanorods (AuNRs) have emerged as promising contrast agents for PAT of vasculature and tumors due to their high signal strength. However, their clinical utility is limited by the overlap between the peak wavelength of their localized surface plasmon resonance and that of hemoglobin. Typically, AuNRs that absorb in the NIR-II region possess a relatively high aspect ratio (∼6), and are grown using a seed-mediated approach. However, their dimensions (80–150 nm length and 12–25 nm width) limit their clinical application. Chen *et al* overcame this limitation by using a hydroquinone seedless method to produce long-aspect-ratio AuNRs, with careful adjustments to NaBH_4_ concentration and pH to increase anisotropy. To enhance PA signal generation, the laser fluence was intentionally set slightly above the damage threshold of the small AuNRs, compensating for the light decay and diffusion within the skin. A pulsed laser with a wavelength of 1,064 nm and a fluence of 25 mJ cm^−2^, along with a needle hydrophone with a central frequency of 20 MHz, were employed to achieve this optimization. Visualization of the PA signals within the murine tumors was achieved using a Vevo 2100 (VisualSonics, Inc.) with a 21 MHz array-US transducer. These miniature gold nanorods produced approximately 3.5 times the PA signal of conventional AuNRs, as shown in figure 8(b), making them superior contrast agents for PAT [139].

Exogenous contrast agents address the inadequacies of endogenous agents via selectively binding to specific molecular targets *in vivo* as well as producing more PA signal when imaged, if desired [2, 92, 93]. Together, these improvements reduce the occurrence of off-target imaging by concentrating within cancer tumors or on the surface of specific cells [92]. Additionally, they can allow for enhanced noise reduction by producing a stronger signal when imaged, making it easier and faster to collect the required data [23, 96]. Unfortunately, these exogenous agents can pose safety risks when used *in vivo*. Nanoparticles that include heavy metals pose some risk for nephrotoxicity or hepatotoxicity if proper steps are not taken to mitigate their accumulation in these organs [143]. Likewise, with every new exogenous agent, much study must be undertaken to ensure it does not irreparably harm the organism which it is designed to image.

## 4 Conclusion

Endogenous and exogenous contrast agents are both essential for advancing the capabilities of PAT, with each class offering distinct advantages that cater to different imaging needs. Endogenous contrast agents, such as hemoglobin, melanin, and lipids, enable noninvasive imaging of structures and functions thanks to their natural biocompatibility. Hemoglobin, in particular, is crucial for visualizing vascular structures and oxygenation, with applications in detecting tumors, monitoring microvascular hemodynamics, and assessing bone healing. Exogenous contrast agents, including proteins, organic dyes, and nanoparticles, offer enhanced specificity and sensitivity. Protein-based agents, such as photoswitchable phytochromes and genetically encoded reporters, enable targeted imaging with customizable absorption properties, improving imaging contrast and allowing functional imaging of biological processes like calcium signaling in neurons. Organic dyes, especially those that absorb in the NIR regions, offer deeper tissue penetration and improved PTT efficacy. Activatable probes, triggered by reactive species or enzymes, further enhance the target-to-background ratio and limit of detection, making them ideal for early disease detection and monitoring therapeutic responses. Nanoparticles, with their tunable optical properties and functional surface modifications, provide even greater versatility for PAT. GNPs, for instance, can be engineered to target specific receptors, like integrin *α*_v_*β*_3_ in neovascularization, or to release therapeutic agents, such as nitric oxide, in a controlled manner. Their high absorption cross-sections and strong PA signals make nanoparticles particularly useful for imaging vasculature, tumors, and retinal vessels, with potential applications in both research and clinical settings.

## Data Availability

All data that support the findings of this study are included within the article (and any supplementary files).
